# From Three-Dimensional (3D)- to 6D-Printing Technology in Orthopedics: Science Fiction or Scientific Reality?

**DOI:** 10.3390/jfb13030101

**Published:** 2022-07-21

**Authors:** Angelo V. Vasiliadis, Nikolaos Koukoulias, Konstantinos Katakalos

**Affiliations:** 1 2nd Orthopedic Department, General Hospital of Thessaloniki “Papageorgiou”, 56403 Thessaloniki, Greece; 2 School of Medicine, Aristotle University of Thessaloniki, 54124 Thessaloniki, Greece; 3 Orthopedic Department, Sports Injuries Unit, Saint Luke’s Hospital, 55236 Thessaloniki, Greece; nkoukoulias@yahoo.gr; 4 Laboratory for Strength of Materials and Structures, Department of Civil Engineering, Aristotle University of Thessaloniki, 54124 Thessaloniki, Greece; kkatakal@civil.auth.gr

**Keywords:** 3D printing, 4D printing, 5D printing, 6D printing, applications, orthopedics

## Abstract

Over the past three decades, additive manufacturing has changed from an innovative technology to an increasingly accessible tool in all aspects of different medical practices, including orthopedics. Although 3D-printing technology offers a relatively inexpensive, rapid and less risky route of manufacturing, it is still quite limited for the fabrication of more complex objects. Over the last few years, stable 3D-printed objects have been converted to smart objects or implants using novel 4D-printing systems. Four-dimensional printing is an advanced process that creates the final object by adding smart materials. Human bones are curved along their axes, a morphological characteristic that augments the mechanical strain caused by external forces. Instead of the three axes used in 4D printing, 5D-printing technology uses five axes, creating curved and more complex objects. Nowadays, 6D-printing technology marries the concepts of 4D- and 5D-printing technology to produce objects that change shape over time in response to external stimuli. In future research, it is obvious that printing technology will include a combination of multi-dimensional printing technology and smart materials. Multi-dimensional additive manufacturing technology will drive the printing dimension to higher levels of structural freedom and printing efficacy, offering promising properties for various orthopedic applications.

## 1. Introduction

Additive manufacturing (AM) is an innovative technology which provides new avenues for the design and manufacture of medical implements, playing an indispensable role in modern medicine [1]. In recent years, additive manufacturing, commonly known as three-dimensional (3D) printing technology, has attracted increasing interest due to its benefits in fabricating microstructure and custom-made medical products through the printing of patient-specific anatomic devices [1,2]. Using a computer-aided design (CAD) package, models can be printed in a layer-by-layer fashion in order to create precise geometric 3D objects with high precision [1,2,3]. There are several different AM techniques, and each vary in the way they form plastic and metal parts, differing in material selection, surface, durability and manufacturing speed and cost [1]. The most commonly used techniques of AM include fused deposition modeling (FDM), powder bed fusion (PBF) and stereolithography (SLA), but there are also other techniques that have been recently developed, such as inkjet printing, cold spraying, friction stirring and diode-based processes [4,5].

## 2. From 3D to 6D Printing Technology

AM has earned an excellent reputation for its applications in the field of biomedical engineering in the past 30 years [1,2]. The concept of 3D systems was introduced in 1984 by Chuck Hull, who is considered the father of 3D printing and was the first to introduce 3D printers using the SLA technique [2]. The applications of 3D printing in orthopedics are also increasingly useful in many aspects, such as in residency training and pre-operative surgical planning, as well as for surgical instruments, personalized bioimplants and patient-specific or customized prostheses [3,6]. Specifically, the 3D-printing facilities in orthopedic surgery have been investigated since the early 2000s, giving promising results. Currently, 3D printing is widely used in complex trauma cases [7], for the management of common childhood orthopedic disorders [8,9], in arthroplasty or extensive reconstructive procedures [10,11], as well as for the treatment of complicated cases in orthopedic oncology [12].

The concept of 4D-printing technology was initially introduced in 2012, showing great potential for application in the fields of aerospace, engineering, automobiles, manufacturing, medicine and many others [13]. Four-dimensional printing is an upgraded version of the commonly used 3D printing which introduces time as the fourth dimension [13,14]. Fundamentally, the aim of 4D printing is to reconstruct the shape of an object with the use of smart materials (SMs), which can change their properties or shapes in response to external stimuli, such as mechanical, chemical, thermal or electrical, over time [1,14,15]. In orthopedics, the main purposes of these upgraded printing technologies are to manufacture specific tools and devices, intelligent tissue-engineered scaffolds which can release drugs or/and cells, and smart orthopedics implants which can change their shape after being implanted in the patient’s body [15,16].

At the moment, the typical shape memory behavior of these smart implants can be used for spinal deformities, fracture fixation, joint replacement and other related orthopedics applications [15,17]. This technology provides new strategies for bone-tissue engineering. One of the major challenges in orthopedics is developing artificial bones and implants that can grow as the child grows and develops [18]. Typical medical implants may not always be suitable for children, as most of them have a fixed size and cannot expand during the child’s natural growth. In order to address this issue, 4D-printed implants can react to different stimuli and change their structure with the passage of time [15,18]. Despite the plethora of advantages, such as the ability to manufacture intelligent materials and the construction of complex anatomical structures [18,19], one of its major potential drawbacks is the deformation control accuracy, due to the fact that the shape-morphing system relies on a biaxial stretch device [20].

To overcome this problem, 5D printing, a new branch of additive manufacturing, was introduced in 2016 by Mitsubishi Electronic Research Laboratories, in which the printing process of a 5D printer can be performed in more axes (containing five degrees of freedom for creating the final object) [1,21]. In this technology, the printer head has the ability to move around from five different angles, while the printed part moves and rotates at defined angles [1,21,22]. Robotic arms have the benefits of speed, agility and flexibility in printing, and can be used as additional devices in order to obtain the final printed object [22]. The main advantage of this technology is the fact that it enables the creation of an object with curved surfaces and improved strength, leading to more well-designed products [21]. Human bones are not flat but have a characterized curved shape, so there is a clear need to manufacture artificial bones with the assistance of 5D-printing technology. In this sense, the 5D-printing system indicates an efficient way to help patients recover from bone fractures and contributes to the restoration of massive and/or complicated bone defects.

The main difference between 3D- and 5D-printing technology is that 5D printing can build an object in five directions (the printer head, which can be a robotic arm, has the ability to move around in five different angles, while the print bed can move back and forth on two axes) rather than one point upward. In addition, this technology creates curved layers which are stronger than the traditional 3D-printed flat layers [1]. Apart from that, both technologies use the same input of a 3D computer-aided design (CAD) file, a 3D scanner and 3D printing materials [21,22]. However, 4D printing is different from these two technologies, adding a new dimension, where the structure can change its shape over the time with the use of SMs. These materials are tunable and have responsive thermo-electro-mechanical properties, as well as the ability to self-repair when damaged [18].

**Figure 1:**
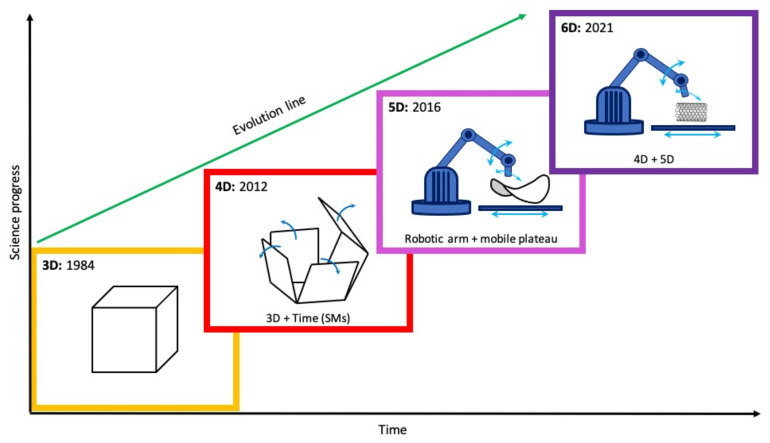
A schematic illustration of 3D-printing technology evolution. The chronological steps of the transformation of 3D-printing technology to the 6D-printing concept.

What about the marriage between 4D- and 5D-printing technology? On the basis of this, for the first time in the international literature, a Greek study makes an attempt to introduce the idea of 6D printing [22]. This means that the printing process will use five degrees of freedom to create the final object, while the final printed structure will be a smart one. It will be expected to provide even more specialized properties and will be capable of changing its shape in response to an environmental stimulus (Figure 1). From an engineer’s point of view, the main benefit of this method is the fact that it enables the construction of a more well-designed object, which exhibits intelligent behavior with a unique response to external stimulus characteristics due to its structural features (printing technology in combination with SMs) [22].

A possible application of 6D-printing technology in orthopedics could be a “smart cast” for the conservative treatment of bone fractures and congenital talipes equinovarus (clubfoot). A 6D-printed orthopedic cast (manufactured with the use of SMs) will be capable of exerting appropriate mechanical correction loads on specific areas to maintain the optimal alignment of a fractured forearm or a malleolus fracture, and thus could achieve favorable clinical efficacy and patient comfort. The most important fact is that this specific “smart cast” will be capable of changing its shape, creating more space or becoming tighter, according to the presence or absence of an edema in the affected limb. In addition, the application of a unique “smart cast” for the treatment of clubfoot will be very useful, helping to avoid the use of serial casting, according to the Ponseti method. In the Ponseti method, the clubfoot intervention usually starts as early as possible, and in the majority of cases, this technique is initiated immediately after the birth. Subsequently, casting takes about four to five weeks in order to achieve the full correction [23]. During this period, manipulation is performed, and casts are changed weekly in different positions to allow the remodeling of the soft tissues and osseocartilaginous structures. A 6D-printed custom-made cast will be able to change its shape and gently move the foot into the correct position. The whole procedure can be conducted without the necessary change of the cast every week but with the use of only one cast, which will be made of smart materials capable of changing their shape over time. These ideas are first described in the present manuscript.

## 3. 3D, 4D, 5D and 6D Printing: Key Aspects and Differences among Different Printing Technologies

The printing technology is theoretically derived from the process of additive manufacturing and enables the fabrication and rapid manufacturing of objects with complex microstructures [15,20]. Due to the fact of an increased demand for personalization, the worldwide need for 3D printers and the manufacturing of smart objects is expected to thrive in the next decade in the medical field [1]. In the medical field, most of the reviewed literature describes the use of 3D-printing technology for surgical guides, anatomical models, and custom-made implants [24]. This technology is mainly less dependent on traditional processing industries. However, the accuracy and quality of the manufactured objects depend on several factors, though mainly on the material chosen [20,25].

The primary difference between 3D- and 4D-printing technology is that 4D printing is capable of manufacturing a dynamic object which can respond to an external stimulus (Table 1) [15,18]. Clearly, at the heart of this printing technology are smart materials, which are capable of reacting to environmental conditions or stimuli (e.g., mechanical, chemical, electrical and/or magnetic signals) over the passage of time [17,19,20]. For this process, a variety of materials can be used, such as smart polymers (with the ability to remember permanent shapes), smart hydrogels (with the ability to achieve reversible changes of their shape and function on demand) and smart scaffolds [17]. Although 4D-printing technology is in its infancy, considerable attention is gained in the field of tissue and organ engineering [26].

The idea of 5D printing is more different than 3D-and 4D-printing processes because it relies on a moving plateau that allows for the printer head to make many angles from five dimensions and fabricate curved objects (Table 1) [21]. Four-dimensional and 5D-printing technologies are the latest roots of additive manufacturing, originating from 3D printing. While the curved parts from 3D printing technology are created by flat and horizontal layers and have some points of weakness, the same curved parts are printed with the 5D-printing technology in multiple dimensions, preventing the formation of weak points and leading to stronger and more complex structures [21,27].

Six-dimensional printing technology is a new branch of AM that was first introduced by Georgantzinos et al., and it is expected to provide solutions of complex material fabrication, leading to lighter and stronger objects with a higher sensitivity to the relevant external stimuli [22]. The major benefit of 6D printing is that it borrows the ability of 5D-printing technology to create complex structures with the use of a robotic arm while minimizing the needs of the material [22]. However, the 6D-printing setup and process cost is expected to be high. The positive economic influence, in combination with the more massive production enabled by 6D printing, will lead to a decrease in the overall cost production in comparison with 4D-printing technology due to the fact that less material will be used to manufacture stronger and more effective projects.

## 4. Conclusions

Despite the separate possible limitations of 4D and 5D printing technologies, 6D printing has vast potential to revolutionize the world of materials that we cannot even imagine today. In some ways, it is not weird to say that there is an intersection between science fiction and science in this area. This creates great expectations, where scientists must search deeper and apply the acquired knowledge in a broad range of healthcare applications. Although 6D printing is an innovative technology, this technology is not yet commonly used. In particular, with the development and the popularity of this specific technology, it is likely to become widespread in the near future. Combining the power of 4D printing with the power of multi-dimensional additive manufacturing technology, scientists will be able to develop “smart objects” and will drastically increase the whole societal impact of AM in all aspects of orthopedic surgery.

## Figures and Tables

**Table 1:** Comparative analysis between different printing technologies.

Characteristics	3D Printing	4D Printing	5D Printing	6D Printing
Building process	A programmed path fabricates a structure by a layer-by-layer fashion from bottom to top.	4D printing is mainly based on 3D printing.	5D printing produces objects from five directions (the print object moves while the printer head is printing).	6D printing is a combination of 4D and 5D printing.
Materials used	Uses thermoplastic polycarbonate, acrylonitrile styrene, photopolymers, resins.	Uses smart materials, including shape memory polymers (SMPs) and hydrogels.	Uses same materials with 3D printing.	Uses smart materials with single or multiple intelligences.
Object shape and durability	Not good in the manufacturing of complex objects.	Object that changes shape due to external stimuli over time.	Object with a curved layer and three to five times stronger than a 3D-printed object.	Capable of changing its shape or properties.
Applications	Biomedical and pharmacological engineering, anatomical designs, medicine, dentistry, aerospace, electronics, education, entertainment, automobile, fashion, defense.			Higher accuracy in medical devices, constructions, aerospace engineering and the manufacturing industry.

## Data Availability

The data presented in this study are available on request from the corresponding author.
